# Single Nucleotide Polymorphisms of *ALDH18A1* and *MAT2A* Genes and Their Genetic Associations with Milk Production Traits of Chinese Holstein Cows

**DOI:** 10.3390/genes13081437

**Published:** 2022-08-12

**Authors:** Wen Ye, Lingna Xu, Yanhua Li, Lin Liu, Zhu Ma, Dongxiao Sun, Bo Han

**Affiliations:** 1 Department of Animal Genetics and Breeding, College of Animal Science and Technology, National Engineering Laboratory for Animal Breeding, China Agricultural University, Key Laboratory of Animal Genetics, Breeding and Reproduction of Ministry of Agriculture and Rural Affairs, Beijing 100193, China; 2 Beijing Dairy Cattle Center, Beijing 100192, China

**Keywords:** genetic effect, SNP, milk traits, dairy cattle, GS

## Abstract

Our preliminary work had suggested two genes, aldehyde dehydrogenase 18 family member A1 (*ALDH18A1*) and methionine adenosyltransferase 2A (*MAT2A*), related to amino acid synthesis and metabolism as candidates affecting milk traits by analyzing the liver transcriptome and proteome of dairy cows at different lactation stages. In this study, the single nucleotide polymorphisms (SNPs) of *ALDH18A1* and *MAT2A* genes were identified and their genetic effects and underlying causative mechanisms on milk production traits in dairy cattle were analyzed, with the aim of providing effective genetic information for the molecular breeding of dairy cows. By resequencing the entire coding and partial flanking regions of *ALDH18A1* and *MAT2A*, we found eight SNPs located in *ALDH18A1* and two in *MAT2A*. Single-SNP association analysis showed that most of the 10 SNPs of these two genes were significantly associated with the milk yield traits, 305-day milk yield, fat yield, and protein yield in the first and second lactations (corrected *p* ≤ 0.0488). Using Haploview 4.2, we found that the seven SNPs of *ALDH18A1* formed two haplotype blocks; subsequently, the haplotype-based association analysis showed that both haplotypes were significantly associated with 305-day milk yield, fat yield, and protein yield (corrected *p* ≤ 0.014). Furthermore, by Jaspar and Genomatix software, we found that 26:g.17130318 C>A and 11:g.49472723G>C, respectively, in the 5′ flanking region of *ALDH18A1* and *MAT2A* genes changed the transcription factor binding sites (TFBSs), which might regulate the expression of corresponding genes to affect the phenotypes of milk production traits. Therefore, these two SNPs were considered as potential functional mutations, but they also require further verification. In summary, *ALDH18A1* and *MAT2A* were proved to probably have genetic effects on milk production traits, and their valuable SNPs might be used as candidate genetic markers for dairy cattle’s genomic selection (GS).

## 1. Introduction

Milk, cheese, yogurt, and other dairy products can provide good nutrition. Some studies have shown that certain components in milk and dairy products are beneficial for gastrointestinal health and have neutral to beneficial effects on biomarkers of inflammation [1,2]. The protein in milk plays a certain role in strengthening muscle, reducing blood pressure, and helping learning and memory. Trans fatty acids in milk fat may be associated with anticarcinogenic properties in humans, and may decrease tumor growth and the risk of coronary heart disease [1]. In recent years, while China’s economy has developed rapidly and people’s living standards have improved, the emphasis on healthy diet is increasing, leading to the soaring proportion of dairy product consumption [3,4]. Meanwhile, China’s dairy industry is developing steadily and has achieved remarkable results, with the total output of milk from 8.27 million tons in 2000 to 36.83 million tons in 2021 [5] according to the National Bureau of Statistics (National Bureau of Statistics. Available online: http://www.stats.gov.cn/ (accessed on 13 June 2022)).

With the gradual transformation of social demand from “the increase of milk intake” to “the high-quality milk consumption”, people prefer milk with high protein and high fat content [6]. Thus, milk production traits, including 305-day milk yield, fat yield, protein yield, fat percentage, and protein percentage, are the major economic traits in dairy cattle breeding. As these traits are quantitative traits, controlled by micro-effect polygenes, susceptible to environmental influences, it is difficult to achieve significant results quickly through conventional breeding alone [7]. At the same time, due to genetic variations in genes, genetic analysis of complex traits is challenging. With the development of molecular genetics, modern biotechnology, and bioinformatics, the emergence of molecular breeding has provided new technical methods for animal breeding. Since 2009, the United States, Canada, and other dairy developed countries have successively applied genomic selection (GS) to cow breeding, which selects target traits through single nucleotide polymorphism (SNP) markers to improve selection intensity, efficiency, and accuracy [8]. Studies have shown that SNPs in the candidate functional genes can significantly affect the milk production traits of dairy cattle [9,10,11,12,13]. Moreover, some researchers showed that extending SNP marker data by adding functional gene information with a large genetic effect of target traits can improve the accuracy of genome breeding value prediction [14,15,16]. Therefore, it is very important for dairy cattle GS to mine the functional genes of target traits and verify the genetic effect of their polymorphic loci. So far, many candidate genes or polymorphisms within these genes have been identified that have a positive correlation with milk production traits in dairy cattle [17]. For instance, a non-conservative substitution of lysine by alanine (K232A) in the diacylglycerol O-acyltransferase 1 (*DGAT1*) gene was found to produce a strong effect on milk composition and yield [18,19,20].

We previously conducted transcriptome and proteome sequencing of liver tissues at different stages of lactation in dairy cows based on the second-generation high-throughput sequencing technology, and identified nine candidate functional genes related to milk production traits, including aldehyde dehydrogenase 18 family member A1 (*ALDH18A1*) and methionine adenosyltransferase 2A (*MAT2A*), which were related to amino acid biosynthesis and metabolism [21]. The vertebrate *ALDH18A1* gene encodes a bifunctional ATP- and NADPH-dependent mitochondrial enzyme with γ-glutamyl kinase and γ-glutamyl phosphate reductase activity, designated as delta 1-pyrroline-5-carboxylate synthase [22]. The enzyme catalyzes the conversion of glutamate to glutamyl semialdehyde, which plays key roles in the regulation of proline, ornithine, and arginine biosynthesis in the body. Ornithine and arginine are key intermediates in the synthesis of urea, creatine, nitric oxide, polyamines, and proteins [23]. *MAT2A* encodes the catalytic subunit α2 to modulate the activity of isoenzyme methionine adenosyltransferase (MAT) with the assistance of regulatory subunit β encoded by methionine adenosyltransferase 2B (*MAT2B*) [24,25]. As an essential enzyme, MAT catalyzes the biosynthesis of S-adenosylmethionine from methionine and ATP [25,26]. It has been demonstrated that overexpression of *MAT2A* promotes lipid accumulation and significantly upregulated the levels of adipogenic marker genes including *PPARγ*, *SREBP-1c*, and *aP2* [27]. So far, these two genes’ influence on productive qualities of farm animals barely has been reported. We found the role of these two genes in milk protein synthesis for the first time in cows in past experiments [21]. In addition, *ALDH18A1* and *MAT2A* genes were located on chr26:26.1094 and chr11:53.7354, and were found to be located within 0.78–4.05 cM and 0.84 cM of quantitative trait locus (QTL) regions that were confirmed to have large genetic effects on milk yield and composition traits, respectively. They were also near to some significant SNPs for milk traits, such as ARS-BFGL-NGS-17995 with the distance of 0.29 Mb from *ALDH18A1*. These data indicate that *ALDH18A1* and *MAT2A* may be involved in milk production traits [21]. The objective of this study was to analyze the genetic effects of the two candidate genes, *ALDH18A1* and *MAT2A*, on 305-day milk yield, fat yield, fat percentage, protein yield, and protein percentage, and we found significant SNPs to provide some reference information for their application on GS chip development in dairy cattle. In addition, we predicted the possible effect of the variants at the identified SNP loci on gene expression based on their location, such as transcription factor binding sites (TFBSs), laying a research foundation for further exploring the causal mutations of important traits in dairy cows.

## 2. Materials and Methods

### 2.1. Animal Selection, Pedigree, and Phenotypic Data Collation

We used 924 daughters of 44 Chinese Holstein bull families from 22 farms of Beijing Shounong Animal Husbandry Development Co., Ltd. (Beijing, China) as the experimental population. Among them, the average number of daughters per bull mentioned above was 21 (ranging from 6 to 62), and each cow had three generations of genealogical information. The offspring were all raised from 2009 to 2015, with good health, accurate pedigree, and standardized Dairy Herd Improvement (DHI) records. The DHI measurement method referred to the national standard of “Technical Specification of Chinese Holstein cattle performance test” (standard number NY/T 1450-2007), and the measurement indicators included 305-day milk yield, fat yield, fat percentage, protein yield, and protein percentage (Table S1). The study was conducted in accordance with Guide for the Care and Use of Laboratory Animals and approved by the Institutional Animal Care and Use Committee (IACUC) at China Agricultural University (Beijing, China; permit number: DK996).

### 2.2. Genomic DNA Extraction from Frozen Semen and Blood Samples

The genomic DNA of the 44 Chinese Holstein bulls’ frozen semen samples and their 924 daughters’ blood samples were extracted by the optimized high-salt method and a TIANamp Blood DNA Kit (Tiangen Biochemical Technology, Beijing, China), respectively. The concentration and integrity of DNA samples were tested using a NanoDrop2000 spectrophotometer (Thermo Science, Hudson, NH, USA) and gel electrophoresis (1.5%), respectively.

### 2.3. Polymorphism Detection of Candidate Genes

Based on the sequence of full coding region and 2000 bp of upstream and downstream regulatory regions of the *ALDH18A1* and *MAT2A* genes of the cattle in GenBank (National Library of Medicine. Available online: https://www.ncbi.nlm.nih.gov/nuccore (accessed on 8 September 2021)), we designed primers (Table S2) using Primer 3.0 (Primer 3. Available online: https://bioinfo.ut.ee/primer3-0.4.0/ (accessed on 8 September 2021)). Then, we diluted the concentration of genomic DNA in the frozen semen to 50 ng/μL, and pipetted 1 μL each into a mixing pool for PCR amplification (Table S2). After the PCR amplification products were tested by 2% gel electrophoresis, the qualified PCR products were sent to Beijing Qingke Xinye Biotechnology Co., Ltd. (Beijing, China) for bidirectional sequencing. Based on the sequencing results, we used the Chromas 1.62 software to view the sequencing diagram and compared the sequence to the reference sequence (ARS-UCD1.2) on NCBI-BLAST (National Library of Medicine. Available online: https://blast.ncbi.nlm.nih.gov/Blast.cgi (accessed on 20 November 2021)) in order to identify polymorphic sites and mutation types. Subsequently, we performed SNP genotype testing on 924 individuals using Genotyping by Target Sequencing (GBTS) technology by Boruidi Biotechnology Co., Ltd. (Hebei, China). The allele and genotype frequencies were directly calculated. The Hardy−Weinberg equilibrium was tested by the chi-squared test to compare the observed genotype frequencies within the expected frequencies.

### 2.4. Linkage Disequilibrium (LD) Estimation

The extent of LD between the identified SNPs was estimated using Haploview 4.2 (Broad Institute of MIT and Harvard, Cambridge, MA, USA). The extent of LD is measured by the D′ value, which is proportional to it. The haplotype block with a frequency greater than 0.05 was retained.

### 2.5. Association Analysis of Single Marker/Haplotype and Milk Production Traits

The MIXED process in SAS 9.4 software (SAS Institute Inc., Cary, NC, USA) was used to conduct association analysis on five milk production traits and SNPs or haplotype blocks, and the animal model used was as follows:

y = µ + HYS + b × M + G + a + e

where Y is the phenotype values of individual milk production traits (305-day milk yield, fat yield, fat percentage, protein yield, and protein percentage); μ is the overall mean; hys is the fixed effect of farm, calving year, and calving season; M is the age of calving as a covariant; b is the regression coefficient of the covariate M; G is the genotype or haplotype combination effect; a is the individual random additive genetic effect, where the distribution is $N\;\left(0,\;A\delta_{a}^{2}\right)$, A is a pedigree-based relationship matrix, and the additive genetic variance is $\delta_{a}^{2}$; and e is the random residual, where the distribution is $N\;\left(0,\;I\delta_{e}^{2}\right)$, the unit matrix is I, and the residual variance is $\delta_{e}^{2}$. We used the Bonferroni multiple test to correct the *p* value of the hypothesis test in the correlation analysis, and obtained the corrected p.

In addition, we calculated the additive, dominant, and substitution effects of SNP loci, using the following formula:

a = (AA − BB )/2, d = AB − (AA + BB)/2, α = a + (q − p) × d

where, a, d, and α are the additive effect, dominant effect, and substitution effect, respectively; AA, AB, and BB are the phenotypic least squares means of the corresponding genotypes; p is the frequency of allele A; and q is the frequency of allele B.

### 2.6. Biological Function Prediction

We used Jaspar (JASPAR. Available online: http://jaspar.genereg.net/ (accessed on 10 April 2022)) and Genomatix (Genomatix software suite. Available online: https://www.genomatix.de/cgi-bin/sessions/login.pl?s=6de98bdc4d8464e81dfaf67276f8f5f7 (accessed on 10 April 2022)) software to predict whether SNPs in the 5′ flanking region *ALDH18A1* and *MAT2A* genes changed the TFBS. Based on the characteristics of the two software, we screened the prediction results by different criteria, that is, the relative score of the former was higher than 0.8, and the binding site of the latter was a highly conservative core sequence. We selected the transcription factors that the two software suggested in order to improve the reliability of the predictions.

## 3. Results

### 3.1. SNPs Identification

We found eight and two SNPs in the *ALDH18A1* and *MAT2A* genes without novel SNPs, respectively. In *ALDH18A1*, 26:g.17130318C>A (rs1091244300) was located in the 5′ flanking region, 26:g.17102977T>C (rs136218403) in intron, 26:g.17118244G>A (rs133295794), 26:g.17100534G>T (rs110706194), and 26:g.17089560G>A (rs458180458) in exon, 26:g.17088978A>G (rs41255559) in the 3′ untranslated region (UTR), and 26:g.17088098G>C (rs208352883) and 26:g.17086802T>A (rs109201383) in the 3′ flanking region. In *MAT2A*, 11:g.49472723G>C (rs109079969) was located in 5′ flanking region and 11:g.49465032C>T (rs110124316) in 3′ flanking region. The genotypic and allelic frequencies of all the identified SNPs are summarized in Table 1.

### 3.2. Single Marker Association Analysis

The associations between the 10 SNPs of the *ALDH18A1* and *MAT2A* genes and 5 milk production traits in the first and second lactation stages, including 305-day milk yield, fat yield, fat percentage, protein yield, and protein percentage, were analyzed. The results showed that for *ALDH18A1*, 26:g.17100534G>T was significantly associated with the 305-day milk yield in the first lactation (corrected *p* = 0.0016; Table 2); 26:g.17130318C>A, 26:g.17118244G>A, 26:g.17102977T>C, 26:g.17100534G>T, and 26:g.17086802T>A reached significant association levels for the 305-day milk, fat, and protein yields in the second lactation (corrected *p* ≤ 0.0488); and 26:g.17088978A>G was significantly associated with the 305-day milk yield, fat yield, fat percentage, and protein yield in the second lactation (corrected *p* ≤ 0.0072). In *MAT2A*, 11:g.49465032C>T was significantly associated with the 305-day milk yield and protein yield in the first lactation (corrected *p* ≤ 0.0252), and the 305-day milk yield, fat yield, and protein yield in the second lactation (corrected *p* ≤ 0.0204), while 11:g.49472723G>C was significantly associated with the 305-day milk yield and protein percentage in the first lactation (corrected *p* ≤ 0.0222). The results of additive, dominant, and substitution effects are shown in Table S3.

### 3.3. Haplotype Association Analysis

**Figure 1:**
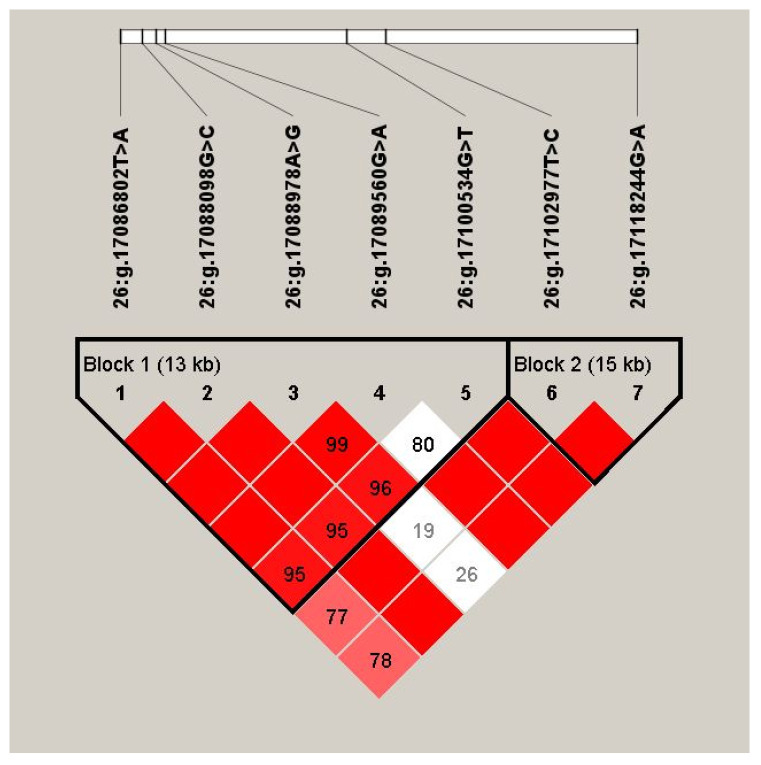
Linkage disequilibrium estimated between SNPs in *ALDH18A1* gene. The blocks indicate haplotype blocks and the text above the horizontal numbers is the SNP names. The values in the red boxes are pair-wise SNP correlations (D′, while bright red boxes without numbers indicate complete Linkage Disequilibrium (LD) (D′ = 1).

Through estimating the degree of LD among the 10 identified SNPs by Haploview 4.2, we discovered that 7 SNPs of the *ALDH18A1* gene formed two haplotype blocks (Figure 1; Block 1: D′ = 0.80–1.00; Block 2: D′ = 0.98–1.00). In Block 1, six haplotypes were found, and the frequencies of H1 (ACGGG), H2 (TGGGG), H3 (TGAAG), H4 (TGAGG), H5 (TGAGT), and H6 (AGGGG) were 0.58, 0.172, 0.094, 0.066, 0.066, and 0.02, respectively. Block 2 consisted of two haplotypes, H1 (TG) and H2 (CA), with frequencies of 0.874 and 0.124, respectively. Moreover, we found that the haplotype combinations had significant associations with the 305-day milk yield, fat yield, fat percentage, protein yield, or protein percentage in the two lactations (corrected *p* ≤ 0.025; Table 3). Block 1 was significantly associated with the 305-day milk yield, fat yield, and protein yield in both lactation stages (corrected *p* ≤ 0.014). Block 2 had a significant association with the 305-day milk yield, fat yield, and protein percentage in the second lactation stage (corrected *p* ≤ 0.025).

### 3.4. Functional Variation Prediction Caused by SNPs

We predicted the TFBS changes of the two SNPs, 26:g.17130318C>A and 11:g.49472723G>C, located in the 5′ region of the *ALDH18A1* and *MAT2A* genes, respectively, using the Jaspar and Genomatix software to obtain the common result (Table 4). Mutation from the allele C to A of 26:g.17130318C>A in *ALDH18A1* caused the disappearance of the binding site (BS) for transcription factor (TF) homeobox containing 1 (HMBOX1) (relative score = 0.92). For 11:g.49472723G>C in *MAT2A*, allele C created the BS for MYC associated zinc finger protein (MAZ) (relative score = 0.84).

## 4. Discussion

Our previous study considered the *ALDH18A1* and *MAT2A* genes to be candidates to affect milk production traits in dairy cows [21]. In this study, we detected the polymorphisms of the *ALDH18A1* and *MAT2A* genes and found that there was a certain genetic association between the SNPs/haplotype blocks and milk production traits. Haplotype analysis has been shown to have a more accurate advantage for detecting genetic variation in complex traits than single-label SNP analysis [28,29].

With different application purposes, gene chips could be divided into high-density gene chips with research value and low-density gene chips with application value [30]. Based on the results of this study, we speculated that there were two possible applications. On the one hand, the reliability of genome breeding value estimation could be improved to a certain extent by increasing the weight of SNPs/ haplotype chromosomal segments of these two genes on high-density SNP chips. On the other hand, considering cost-effectiveness, low-density gene chips can also be constructed by selecting loci such as SNPs of *ALDH18A1* and *MAT2A* that are closely associated with milk production traits, focusing on estimating the breeding value of the target traits [30,31]. At present, many studies use low-density SNP chipsets to genotype animals, and then accurately interpolate them into high-density groups to improve the accuracy of breeding value estimation [32,33,34]. However, the application in this direction still needs in-depth verification.

Furthermore, for each identified SNP of *ALDH18A1* and *MAT2A* in the current study, we observed that the phenotypic value of individuals with low-frequency alleles is low, which may indicate that due to long-term artificial breeding of high-yield individuals, the proportion of genotypes that have a beneficial impact on the phenotype gradually occupies a large proportion. This implied the effectiveness of dairy cattle breeding in recent years, and also reflected that these loci might be closely associated with milk production traits. Moreover, low-frequency variants (such as 26:g.17100534G>T) may be caused by potential sampling error effects. If not, we need to increase sample size to vigorously identify such associations, which has been emphasized by recent studies that identified the role of low frequency and rare coding variation in complex traits [35]. However, we found that the ten SNPs of *ALDH18A1* and *MAT2A* showed different associations between the first and second lactations, and considered that it might be caused by the different number of cows selected for genetic association analysis. As we used 924 cows in the first lactation and 635 in the second lactation (the cows selected in the two lactation periods did not completely overlap), the statistical significance might have been impacted. Generally, cows have higher milk production in the second lactation, and the different physiologic status of cows between the two lactations was also one possibility for the different genetic effects across lactations. In addition, the results showed that for some SNPs, there were an increased value in the heterozygous genotype, whereas there were others with an increased value in the homozygous genotype. This might have been caused by the interaction between alleles. Taking the milk production in the second lactation period as an example, for 26:g.17118244G>A, the phenotypic value of heterozygotes was the highest because of the significant dominant effect; for 26:g.17086802T>A, the phenotypic value of heterozygotes was in the middle because of the significant additive effect.

There are many factors that affect gene expression, one of which is that TFBSs regulate the transcription of target genes by binding to different transcription factors [36].The SNPs located at TFBSs may affect the binding of transcription factors, resulting in differences in gene expression among individuals with different genotypes [37]. In this study, we found the changes of TFBSs caused by the SNPs in the 5′ flanking regions of the *ALDH18A1* and *MAT2A* genes. In *ALDH18A1,* the BS of TF HMBOX1 appeared when the allele A mutated to C of 26:g.17130318C>A. HMBOX1 is a homeobox containing protein with transcriptional repressor activity [38,39,40]. Based on the phenotypic data of milk production traits with different genotypes, we found that, for 26:g.17130318C>A in *ALDH18A1*, the fat and protein yields of cows with genotype AA were significantly higher than those with genotype CC in the second lactation. These findings suggest that the mutant site 26:g.17130318C>A could modulate the expression of *ALDH18A1* to affect the milk yield traits in dairy cattle by binding the TF HMBOX1. In addition, for 11:g.49472723G>C in *MAT2A*, allele C created the BS for MAZ. MAZ is a transcription factor that plays a dual regulatory role in initiating and terminating the transcription of certain genes [41,42,43,44]. The 305-day milk yield and protein percentage of CC genotype individuals at 11:g.49472723G>C in *MAT2A* were significantly lower than those of GG individuals in the first lactation. The above findings indicate that 11:g.49472723G>C might be a key mutation that affects the phenotypic changes of milk production by the regulation of *MAT2A* gene by binding the TF MAZ. Thus, we speculated that the change of gene expression caused by SNPs may be one of the reasons for the phenotypic changes of milk production traits in dairy cows.

Furthermore, this study has some limitations compared with a genome-wide association study (GWAS). GWAS can screen SNPs in the whole genome and find parts associated with traits. At the same time, systematic errors such as population stratification can be corrected in the analysis process. Therefore, the significant SNPs obtained from GWAS are generally more reliable than those obtained from allelic association. Indeed, GWAS gives the possibility to take into account a massive amount of SNPs; however, most of them have no influence on production traits. SNPs with no impact on production traits can cause information noise. Moreover, each SNP found using a high-density microarray should be analyzed in detail to prove the associations with production traits.

## 5. Conclusions

This study identified single nucleotide polymorphisms of the *ALDH18A1* and *MAT2A* genes, and found that most of these SNPs were associated with milk production traits of cows. We propose that these SNPs could be used as genetic markers for milk production traits in dairy GS; however, this must be confirmed on larger populations of dairy cattle. Two SNPs, 26:g.17130318C>A in *ALDH18A1* and 11:g.49472723G>C in *MAT2A*, might change the TFBSs to regulate expression of the corresponding gene. This study also provided some reference information for further functional verification of *ALDH18A1* and *MAT2A*.

## Figures and Tables

**Table 1:** Frequencies of genotypes and alleles of identified SNPs in aldehyde dehydrogenase 18 family member A1 (*ALDH18A1*) and methionine adenosyltransferase 2A (*MAT2A*) genes.

Gene	SNP Name	RS ID	Position (UMD3.1)	Gene Region	Genotype	Genotypic Frequency	Allele	Allelic Frequency	Hardy–Weinberg Equilibrium
*ALDH18A1*	26:g.17130318C>A	rs109124430	Chr26:	5′ flanking region	AA	0.6840	A	0.8274	T
			17130318		AC	0.2868	C	0.1726	
					CC	0.0292			
	26:g.17118244G>A	rs133295794	Chr26:	Exon (synonymous)	AA	0.0195	A	0.1239	T
			17118244		AG	0.2089	G	0.8761	
					GG	0.7716			
	26:g.17102977T>C	rs136218403	Chr26:	Intron	CC	0.0216	C	0.1261	T
			17102977		CT	0.2089	T	0.8739	
					TT	0.7695			
	26:g.17100534G>T	rs110706194	Chr26:	Exon (synonymous)	GG	0.8680	G	0.9324	T
			17100534		GT	0.1288	T	0.0676	
					TT	0.0032			
	26:g.17089560G>A	rs458180458	Chr26:	Exon (synonymous)	AA	0.0119	A	0.0947	T
			17089560		AG	0.1656	G	0.9053	
					GG	0.8225			
	26:g.17088978A>G	rs41255559	Chr26:	3′UTR (untranslated region)	AA	0.0606	A	0.2262	T
			17088978		AG	0.3312	G	0.7738	
					GG	0.6082			
	26:g.17088098G>C	rs208352883	Chr26:	3′ flanking region	CC	0.3506	C	0.5812	T
			17088098		CG	0.4610	G	0.4188	
					GG	0.1883			
	26:g.17086802T>A	rs109201383	Chr26:	3′ flanking region	AA	0.3712	A	0.6012	T
			17086802		AT	0.4600	T	0.3988	
					TT	0.1688			
*MAT2A*	11:g.49472723G>C	rs109079969	Chr26:	5′ flanking region	CC	0.6396	C	0.8003	T
			49472723		CG	0.3214	G	0.1997	
					GG	0.0390			
	11:g.49465032C>T	rs110124316	Chr26:	3′ flanking region	CC	0.4913	C	0.6813	F
			49465032		CT	0.3798	T	0.3187	
					TT	0.1288			

Note: T indicates that the gene and genotype frequencies conform to Hardy–Weinberg equilibrium law, while F indicates that they do not.

**Table 2:** Associations of 10 SNPs in *ALDH18A1* and *MAT2A* genes with milk yield and composition traits in Chinese Holstein cattle during first and second lactations.

Genes	SNPs	Lactation	Genotype (No.)	Milk Yield (kg)	Fat Yield (kg)	Fat Percentage (%)	Protein Yield (kg)	Protein Percentage (%)
*ALDH18A1*	26:g.17130318C>A	1	AA (632)	10,312 ± 59.78	339.4 ± 2.66	3.31 ± 0.02	305.9 ± 1.94	2.98 ± 0.02
			AC (265)	10,145 ± 69.33	338.84 ± 3.01	3.37 ± 0.03	302.59 ± 2.19	3.00 ± 0.02
			CC (27)	10,211 ± 149.06 ^a^	339.89 ± 6.11	3.36 ± 0.06	305.07 ± 4.45	3.00 ± 0.04
			$\chi^{2}$	8.76	0.07	6.86	3.92	2.15
			*p* value	0.0128	0.8714	0.0329	0.1405	0.3414
			Corrected *p*	0.1024	6.9712	0.2632	1.124	2.7312
		2	AA (437)	10,850 ± 61.29	388.69 ± 2.73 ^Aa^	3.59 ± 0.03	322 ± 1.99 ^Aa^	2.97 ± 0.02
			AC (181)	10,694 ± 76.68	383.71 ± 3.31 ^Aa^	3.59 ± 0.03	315.51 ± 2.41 ^ABb^	2.95 ± 0.02
			CC (17)	10,331 ± 194.55	349.48 ± 7.96 ^Bb^	3.40 ± 0.08	299.99 ± 5.80 ^Bb^	2.91 ± 0.05
			$\chi^{2}$	10.81	26.7	6.09	22.03	3.14
			*p* value	0.0047	<0.0001	0.0485	<0.0001	0.2088
			Corrected *p*	0.0376	<0.0001	0.388	<0.0001	1.6704
	26:g.17118244G>A	1	AA (18)	10,232 ± 179.35	344.82 ± 7.31	3.39 ± 0.07	304.58 ± 5.33	2.99 ± 0.04
			AG (193)	10,292 ± 75.23	342.37 ± 3.24	3.36 ± 0.03	306.49 ± 2.36	3.00 ± 0.02
			GG (713)	10,251 ± 58.52	338.26 ± 2.62	3.32 ± 0.02	304.47 ± 1.90	2.98 ± 0.02
			$\chi^{2}$	0.46	3.36	2.64	1.24	0.76
			*p* value	0.796	0.187	0.2675	0.5381	0.6851
			Corrected *p*	6.368	1.496	2.14	4.3048	5.4808
		2	AA (12)	10,527 ± 227.41 ^ab^	362.45 ± 9.26 ^a^	3.46 ± 0.09	307.38 ± 6.75	2.93 ± 0.05
			AG (133)	10,997 ± 84.80 ^a^	391.85 ± 3.63 ^b^	3.57 ± 0.03	325.40 ± 2.64	2.96 ± 0.02
			GG (490)	10,751 ± 60.15 ^b^	385.76 ± 2.69 ^ab^	3.59 ± 0.02	318.67 ± 1.96	2.97 ± 0.02
			$\chi^{2}$	11.76	11.5	2.25	12.73	0.71
			*p* value	0.003	0.0034	0.3248	0.0019	0.7024
			Corrected *p*	0.024	0.0272	2.5984	0.0152	5.6192
	26:g.17102977T>C	1	CC (20)	10,240 ± 171.14	343.5 ± 6.98	3.37 ± 0.07	305.74 ± 5.09	3.00 ± 0.04
			CT (193)	10,278 ± 75.15	342.12 ± 3.23	3.36 ± 0.03	306 ± 2.36	3.00 ± 0.02
			TT (711)	10,255 ± 58.58	338.33 ± 2.62	3.32 ± 0.02	304.55 ± 1.91	2.98 ± 0.02
			$\chi^{2}$	0.16	2.73	2.72	0.67	0.8
			*p* value	0.9253	0.2564	0.2577	0.7137	0.6691
			Corrected *p*	7.4024	2.0512	2.0616	5.7096	5.3528
		2	CC (12)	10,524 ± 227.4	362.32 ± 9.26	3.46 ± 0.09	307.29 ± 6.75	2.93 ± 0.05
			CT (134)	10,978 ± 84.37	390.97 ± 3.61	3.57 ± 0.03	324.86 ± 2.63	2.96 ± 0.02
			TT (489)	10,755 ± 60.20	385.96 ± 2.69 ^B^	3.59 ± 0.02	318.78 ± 1.96	2.97 ± 0.02
			$\chi^{2}$	10.11	10.31	2.45	11.22	0.71
			*p* value	0.0067	0.0061	0.2946	0.0039	0.7021
			Corrected *p*	0.0536	0.0488	2.3568	0.0312	5.6168
	26:g.17100534G>T	1	GG (802)	10,306 ± 58.10 ^Aa^	340.22 ± 2.60	3.33 ± 0.02	305.91 ± 1.89	2.98 ± 0.02
			GT (119)	9977.38 ± 87.89 ^Bb^	333.57 ± 3.72	3.38 ± 0.04	298.81 ± 2.71	3.01 ± 0.02
			TT (3)	10,230 ± 421.67 ^ab^	337.7 ± 17.04	3.33 ± 0.17	305.44 ± 12.43	3.00 ± 0.10
			$\chi^{2}$	17.74	4.41	2.48	9.48	2.19
			*p* value	0.0002	0.1109	0.2903	0.009	0.3343
			Corrected *p*	0.0016	0.8872	2.3224	0.072	2.6744
		2	GG (550)	10,878 ± 59.18 ^Aa^	389.39 ± 2.66 ^Aa^	3.58 ± 0.02	322.33 ± 1.93 ^Aa^	2.97 ± 0.02
			GT (82)	10,222 ± 104.36 ^Bb^	366.03 ± 4.39 ^Bb^	3.58 ± 0.04	301.48 ± 3.20 ^Bb^	2.95 ± 0.03
			TT (3)	10,129 ± 433.12 ^ab^	361.25 ± 17.55 ^ab^	3.59 ± 0.17	302.88 ± 12.802 ^ab^	2.97 ± 0.10
			$\chi^{2}$	44.84	34.64	0	51.09	0.58
			*p* value	<0.0001	<0.0001	0.9985	<0.0001	0.7473
			Corrected *p*	<0.0001	<0.0001	7.988	<0.0001	5.9784
	26:g.17089560G>A	1	AA (11)	10,748 ± 231.33	354.55 ± 9.42	3.30 ± 0.09	321.51 ± 6.87	2.98 ± 0.06
			AG (153)	10,285 ± 84.85	339.32 ± 3.63	3.33 ± 0.03	304.86 ± 2.65	2.98 ± 0.02
			GG (760)	10,253 ± 57.75	339.19 ± 2.58	3.33 ± 0.02	304.83 ± 1.88	2.99 ± 0.02
			$\chi^{2}$	4.77	2.9	0.15	6.47	0.22
			*p* value	0.0928	0.2354	0.9278	0.0398	0.8947
			Corrected *p*	0.7424	1.8832	7.4224	0.3184	7.1576
		2	AA (8)	10,490 ± 295.13	378.43 ± 12.00	3.61 ± 0.12	319.19 ± 8.7487	3.03 ± 0.07
			AG (103)	10,640 ± 95.67	378.74 ± 4.06	3.57 ± 0.04	315.39 ± 2.9595	2.97 ± 0.02
			GG (524)	10,827 ± 59.57	387.96 ± 2.67	3.59 ± 0.02	320.55 ± 1.944	2.96 ± 0.02
			$\chi^{2}$	5.16	6.55	0.28	3.73	0.97
			*p* value	0.0766	0.0387	0.8696	0.1559	0.6172
			Corrected *p*	0.6128	0.3096	6.9568	1.2472	4.9376
	26:g.17088978A>G	1	AA (56)	10,161 ± 117.76	341.96 ± 4.89	3.39 ± 0.05	305.17 ± 3.57	3.01 ± 0.03
			AG (306)	10,250 ± 67.89	338.46 ± 2.96	3.33 ± 0.03	305.17 ± 2.16	2.99 ± 0.02
			GG (562)	10,273 ± 60.16	339.35 ± 2.67	3.33 ± 0.02	304.71 ± 1.95	2.98 ± 0.02
			$\chi^{2}$	1.02	0.65	1.71	0.09	1.44
			*p* value	0.6	0.7229	0.4256	0.9564	0.487
			Corrected *p*	4.8	5.7832	3.4048	7.6512	3.896
		2	AA (42)	10,538 ± 139.32 ^AB^	362.98 ± 5.77 ^Aa^	3.42 ± 0.06 ^Aa^	309.2 ± 4.21 ^Aa^	2.93 ± 0.03
			AG (206)	10,581 ± 74.38 ^A^	383.18 ± 3.22 ^Bb^	3.63 ± 0.03 ^Bb^	314.45 ± 2.35 ^Aa^	2.97 ± 0.02
			GG (387)	10,927 ± 63.06 ^B^	390.75 ± 2.80 ^Bb^	3.58 ± 0.03 ^ab^	323.48 ± 2.04 ^Bb^	2.97 ± 0.02
			$\chi^{2}$	27.58	25.8	14.13	25.1	1.74
			*p* value	<0.0001	<0.0001	0.0009	<0.0001	0.4186
			Corrected *p*	<0.0001	<0.0001	0.0072	<0.0001	3.3488
	26:g.17088098G>C	1	CC (324)	10,333 ± 66.66	341.06 ± 2.93	3.32 ± 0.03	305.57 ± 2.13	2.97 ± 0.02
			CG (426)	10,215 ± 62.67	337.21 ± 2.76	3.33 ± 0.03	304.24 ± 2.01	2.99 ± 0.02
			GG (174)	10,225 ± 78.95	341.27 ± 3.38	3.37 ± 0.03	305.25 ± 2.46	3.00 ± 0.02
			$\chi^{2}$	4.57	3.89	2.16	0.73	3.49
			*p* value	0.1025	0.1436	0.3401	0.696	0.175
			Corrected *p*	0.82	1.1488	2.7208	5.568	1.4
		2	CC (233)	10,970 ± 71.62 ^Aa^	396.06 ± 3.13 ^Aa^	3.61 ± 0.03	324.66 ± 2.28 ^Aa^	2.97 ± 0.02
			CG (277)	10,708 ± 68.62 ^ABb^	383.33 ± 3.01 ^Bb^	3.59 ± 0.03	317.99 ± 2.19 ^ABb^	2.97 ± 0.02
			GG (125)	10,641 ± 87.94 ^Bb^	374.74 ± 3.74 ^Bb^	3.52 ± 0.04	314.05 ± 2.73 ^Bb^	2.95 ± 0.02
			$\chi^{2}$	17.34	34.84	6.37	16.71	1.12
			*p* value	0.0002	<0.0001	0.0423	0.0003	0.5723
			Corrected *p*	0.0016	<0.0001	0.3384	0.0024	4.5784
	26:g.17086802T>A	1	AA (343)	10,321 ± 65.81	341.52 ± 2.89	3.33 ± 0.03	305.33 ± 2.11	2.97 ± 0.02
			AT (425)	10,217 ± 62.63	336.33 ± 2.76	3.32 ± 0.03	304.26 ± 2.01	2.99 ± 0.02
			TT (156)	10,234 ± 81.80	343.07 ± 3.49	3.38 ± 0.03	305.75 ± 2.54	3.00 ± 0.02
			$\chi^{2}$	3.65	8.43	4.55	0.73	3.27
			*p* value	0.1615	0.0151	0.1034	0.6944	0.1954
			Corrected *p*	1.292	0.1208	0.8272	5.5552	1.5632
		2	AA (247)	11,016 ± 70.14 ^Aa^	396.72 ± 3.07 ^Aa^	3.60 ± 0.03	325.69 ± 2.23 ^Aa^	2.96 ± 0.02
			AT (272)	10,666 ± 69.12 ^Bb^	382.36 ± 3.03 ^Bb^	3.59 ± 0.03	316.75 ± 2.20 ^Bb^	2.97 ± 0.02
			TT (116)	10,580 ± 90.67 ^Bb^	372.28 ± 3.85 ^Bb^	3.52 ± 0.04	312.89 ± 2.80 ^Bb^	2.96 ± 0.02
			$\chi^{2}$	31.11	45.41	5.8	26.55	0.65
			*p* value	<0.0001	<0.0001	0.0559	<0.0001	0.7242
			Corrected *p*	<0.0001	<0.0001	0.4472	<0.0001	5.7936
*MAT2A*	11:g.49472723G>C	1	CC (591)	10,307 ± 60.23 ^a^	339.22 ± 2.68	3.31 ± 0.02	304.92 ± 1.95	2.97 ± 0.02 ^a^
			CG (297)	10,150 ± 67.98 ^b^	338.26 ± 2.96	3.36 ± 0.03	304.07 ± 2.15	3.01 ± 0.02 ^b^
			GG (36)	10,376 ± 137.72 ^ab^	348.4 ± 5.68	3.39 ± 0.06	311.23 ± 4.14	3.01 ± 0.03 ^ab^
			$\chi^{2}$	9.05	3.54	6.34	3.33	10.16
			*p* value	0.0111	0.1707	0.0426	0.1899	0.0064
			Corrected *p*	0.0222	0.3414	0.0852	0.3798	0.0128
		2	CC (410)	10,829 ± 62.7056	387.57 ± 2.79	3.58 ± 0.03	320.29 ± 2.03	2.96 ± 0.02
			CG (201)	10,707 ± 74.7301	384.71 ± 3.24	3.59 ± 0.03	318.45 ± 2.36	2.97 ± 0.02
			GG (24)	11,006 ± 168.94	383.24 ± 6.93	3.48 ± 0.07	322.02 ± 5.05	2.92 ± 0.04
			$\chi^{2}$	4.96	1.22	2.7	1.05	2.13
			*p* value	0.085	0.5426	0.2596	0.5909	0.3455
			Corrected *p*	0.17	1.0852	0.5192	1.1818	0.691
	11:g.49465032C>T	1	CC (454)	10,313 ± 62.47 ^a^	340.38 ± 2.76	3.33 ± 0.03	306.92 ± 2.01 ^a^	2.99 ± 0.02
			CT (351)	10,167 ± 64.95 ^b^	336.71 ± 2.85	3.34 ± 0.03	302.05 ± 2.07 ^b^	2.98 ± 0.02
			TT (119)	10,327 ± 86.35 ^ab^	342.48 ± 3.66	3.33 ± 0.04	305.58 ± 2.67 ^ab^	2.97 ± 0.02
			$\chi^{2}$	8.8	4.57	0.14	9.83	1.07
			*p* value	0.0126	0.1022	0.9317	0.0075	0.5869
			Corrected *p*	0.0252	0.2044	1.8634	0.015	1.1738
		2	CC (316)	10,914 ± 66.32 ^A^	387.23 ± 2.92 ^ab^	3.55 ± 0.03	323.86 ± 2.13 ^Aa^	2.97 ± 0.02
			CT (235)	10,763 ± 71.46 ^AB^	389.58 ± 3.11 ^a^	3.62 ± 0.03	316.58 ± 2.27 ^Bb^	2.95 ± 0.02
			TT (84)	10,496 ± 99.15 ^B^	376.99 ± 4.17 ^b^	3.60 ± 0.04	314.32 ± 3.04 ^Bb^	3.00 ± 0.03
			$\chi^{2}$	18.81	9.26	6.05	18.36	5.1
			*p* value	<0.0001	0.0102	0.0495	0.0001	0.0791
			Corrected *p*	<0.0001	0.0204	0.099	0.0002	0.1582

Note: The number in the table represents the mean ± standard deviation; the number in the bracket represents the number of cows for the corresponding genotype; *p* value shows the significance for the genetic effects of SNPs; the superscript letters indicate the significance of different genotypes, involving the comparison between each two pairs; a, b within the same column with different superscripts means *p* < 0.05; and A, B within the same column with different superscripts means *p* < 0.01. Corrected *p* means *p* value after multiple test correction of Bonferroni.

**Table 3:** Haplotype analyses for *ALDH18A1* gene.

Block	Lactation	Haplotype Combination	Milk Yield (kg)	Fat Yield (kg)	Fat Percentage (%)	Protein Yield (kg)	Protein Percentage (%)
Block1	1	H1H1 (322)	10,425 ± 67.38 ^a^	344.75 ± 2.98	3.32 ± 0.03	308.87 ± 2.17 ^ab^	2.97 ± 0.02
		H1H2 (174)	10,260 ± 77.58 ^ab^	336.27 ± 3.36	3.30 ± 0.032	305.59 ± 2.45 ^ab^	2.99 ± 0.02
		H1H3 (92)	10,466 ± 96.58 ^a^	343.07 ± 4.10	3.29 ± 0.04	311.62 ± 2.99 ^ab^	2.99 ± 0.03
		H1H4 (68)	10,422 ± 105.67 ^ab^	347.38 ± 4.43	3.34 ± 0.04	313.52 ± 3.23 ^a^	3.02 ± 0.03
		H1H5 (71)	10,097 ± 105.9 ^b^	334.61 ± 4.46	3.34 ± 0.04	301.61 ± 3.25 ^b^	3.00 ± 0.03
		$\chi^{2}$	15.59	14.34	2.01	14.25	4.59
		*p* value	0.0039	0.0067	0.7333	0.007	0.3327
		Corrected *p*	0.0078	0.0134	1.4666	0.014	0.6654
	2	H1H1 (231)	11,060 ± 71.76	398.4 ± 3.15	3.60 ± 0.03	328.22 ± 2.29	2.97 ± 0.02
		H1H2 (108)	10,964 ± 90.72	388.92 ± 3.86	3.55 ± 0.04	327.25 ± 2.81	2.98 ± 0.02
		H1H3 (60)	10,704 ± 115.33	381.95 ± 4.83	3.59 ± 0.05	319.31 ± 3.52	2.99 ± 0.03
		H1H4 (44)	10,447 ± 132.22	379.36 ± 5.51	3.62 ± 0.05	310.99 ± 4.02	2.98 ± 0.03
		H1H5 (50)	10,320 ± 128.01	373.52 ± 5.33	3.62 ± 0.05	307.29 ± 3.89	2.98 ± 0.03
		$\chi^{2}$	50.87	35.23	2.6	47.84	0.53
		*p* value	<0.0001	<0.0001	0.769	<0.0001	0.9707
		Corrected *p*	<0.0001	<0.0001	1.538	<0.0001	1.9414
Block2	1	H1H1 (711)	10,351 ± 58.33	343.72 ± 2.61	3.34 ± 0.02	307.27 ± 1.90	2.97 ± 0.02
		H1H2 (191)	10,342 ± 75.13	343.81 ± 3.24	3.35 ± 0.03	306.99 ± 2.36	2.98 ± 0.02
		$\chi^{2}$	0.03	0	0.39	0.02	0.08
		*p* value	0.8736	0.9716	0.5314	0.879	0.7734
		Corrected *p*	1.7472	1.9432	1.0628	1.758	1.5468
	2	H1H1 (489)	10,804 ± 61.05 ^Aa^	386.12 ± 2.73 ^a^	3.5771 ± 0.03	321.12 ± 1.99 ^Aa^	2.97 ± 0.02
		H1H2 (133)	11,083 ± 83.35 ^Bb^	393.98 ± 3.55 ^b^	3.5669 ± 0.03388	328.79 ± 2.59 ^Bb^	2.96 ± 0.02
		$\chi^{2}$	12.98	6.29	0.11	11.25	0.11
		*p* value	0.0003	0.0125	0.7416	0.0009	0.7456
		Corrected *p*	0.0006	0.025	1.4832	0.0018	1.4912

Note: The number in the table represents the mean ± standard deviation; H means haplotype; in Block1, H1: ACGGG, H2: TGGGG, H3: TGAAG, H4: TGAGG, H5: TGAGT; in Block2, H1: TG, H2: CA; the number in the bracket represents the number of cows for the haplotype combination; *p* value shows the significance for the genetic effects of haplotype combination; the superscript letters indicate the significance of different genotypes, involving the comparison between each two pairs; a, b within the same column with different superscripts means *p* < 0.05; and A, B within the same column with different superscripts means *p* < 0.01. Corrected *p* means *p* value after multiple test correction of Bonferroni.

**Table 4:** Transcription factor binding sites (TFBSs) prediction for SNPs in *ALDH18A1* and *MAT2A* genes.

Gene	SNPs	Allele	Transcription Factor	Relative Score (≥0.80; Jasper)	Predicted Binding Site Sequence (Genomatix)
*ALDH18A1*	26:g.17130318C>A	C	HMBOX1	0.92	ACTAGTTTAG
		A			
*MAT2A*	11:g.49472723G>C	G			
		C	MAZ	0.84	CGCGGCTCCCC

## Data Availability

The datasets generated and/or analyzed during the current study are available in the article and its additional files.

## Supplementary Materials

The following supporting information can be downloaded at: https://www.mdpi.com/article/10.3390/genes13081437/s1, Table S1: Descriptive statistics of phenotypic values for dairy production traits in two lactations; Table S2: Primers and procedures for PCR used in SNPs identification of *ALDH18A1* and *MAT2A* genes; Table S3: Additive, dominant, and substitution effects of SNPs in *ALDH18A1* and *MAT2A* genes on milk yield and composition traits in Chinese Holstein cattle during two lactations.
